# A Plan for Facilitating the Reduction of Strangulated Hernia by Taxis

**Published:** 1872-03

**Authors:** 


### A Plan for Facilitating the Reduction of Strangulated Hernia by Taxis.

Dr. P. C. Smyly, in the *British Medical Journal* for December 25, 1871, reports a plan by which he has successfully reduced strangulated hernia when all other plans had failed. "In inguinal hernia in the male the index finger is applied to the lowest part of the scrotum. This is invaginated, the finger being passed behind the testicle and cord up to the external ring. The hernial tumor is then pressed downwards over the finger, toward the back of the hand, so as to make the strictures in the ring tense, and consequently smaller. The invaginated finger is then forced firmly upwards and outwards in the direction of the internal ring. As soon as the finger is firmly grasped the hand should be slightly turned, and the finger pushed toward the middle line. Considerable force may be safely applied in this way, as all the delicate structures are behind the finger, which acts mainly on the stricture. On withdrawing the finger the hernia can usually be easily returned. The same principle is equally applicable to femoral hernia." The advantages claimed for this plan are: 1st. The strangulating portion of the ring is dilated before any pressure is applied to the bowel. 2d. Much greater force may be applied to dilate than could safely be brought to bear when the intestine is employed for dilatation, as in ordinary taxis. 3d. There is much greater probability of returning the bowel into the abdomen in a good condition, and consequently in a number of cases avoiding a dangerous surgical operation.—*Detroit Review of Medicine.*

